# Soluble PD-1: Predictive, Prognostic, and Therapeutic Value for Cancer Immunotherapy

**DOI:** 10.3389/fimmu.2020.587460

**Published:** 2020-11-19

**Authors:** Muhammad Khan, Zhihong Zhao, Sumbal Arooj, Yuxiang Fu, Guixiang Liao

**Affiliations:** ^1^ Department of Radiation Oncology, Shenzhen People’s Hospital, The First Affiliated Hospital of Southern University of Science and Technology, Shenzhen, China; ^2^ Department of Oncology, First Affiliated Hospital of Anhui Medical University, Hefei, China; ^3^ Department of Nephrology, Shenzhen People’s Hospital, Second Clinical Medicine Centre, Jinan University, Shenzhen, China; ^4^ Department of Biochemistry, University of Sialkot, Sialkot, Pakistan

**Keywords:** soluble programmed cell death protein 1, local gene therapy, cancer vaccination, prediction, prognosis, immune checkpoint blockade

## Abstract

Programmed death protein 1 (PD-1) interaction with PD-L1 deliver immunosuppressive environment for tumor growth, and its blockade with directed monoclonal antibodies (anti-PD-1/anti-PD-L1) has shown remarkable clinical outcome. Lately, their soluble counterparts, sPD-1 and sPD-L1, have been detected in plasma, and elevated levels have been associated with advanced disease, clinical stages, and worst prognosis for cancer patients. Elevated plasma levels of sPD-L1 have been correlated with worst prognosis in several studies and has displayed a persistent outlook. On the other hand, sPD-1 levels have been inconsistent in their predictive and prognostic ability. Pretherapeutic higher sPD-1 plasma levels have shown to predict advanced disease state and to a lesser extent worst prognosis. Any increase in sPD-1 plasma level post therapeutically have been correlated with improved survival for various cancers. *In vitro* and *in vivo* studies have shown sPD-1 ability to bind PD-L1 and PD-L2 and block PD-1/PD-L1 interaction. Local delivery of sPD-1 in cancer tumor microenvironment through local gene therapy have demonstrated an increase in tumor specific CD8+ T cell immunity and tumor growth reduction. It had also exhibited enhancement of T cell immunity induced by vaccination and other gene therapeutic agents. Furthermore, it may also lessen the inhibitory effect of circulating sPD-L1 and enhance the effects of mAb-based immunotherapy. In this review, we highlight various aspects of sPD-1 role in cancer prediction, prognosis, and anti-cancer immunity, as well as, its therapeutic value for local gene therapy or systemic immunotherapy in blocking the PD-1 and PD-L1 checkpoint interactions.

## Introduction

Adaptive immune system comprising T cells (CD 4+ and CD 8+ T cells) and B cells is capable of destroying and eliminating any foreign harmful invader while sparing normal healthy cells through self-tolerance (1). Antigens are presented to T cells by antigen presenting cells (APCs) for elimination through T cells activation, and induction of its effector functions (2, 3). T cell receptor (TCR) binding to MHC-peptide complex presented by APCs, and the costimulatory receptor CD28 to its ligands CD80/86 present on APCs results in T cell stimulation (2–4). In order to avoid overactivation, negative regulators such as CTLA-4 is induced on the T cells (5). CTLA-4 competes for same ligands as CD28 and cause T cell inhibition (6). Programmed cell death protein (PD-1) is also induced after T cell is activated for which ligands on cancer cells and APCs are up-regulated (7, 8). PD-1 and PD-L1 interaction results in inhibition of its effector function such as cytotoxicity and cytokine release, restricts T cell proliferation and survival, and induces apoptotic death of tumor-specific T cells (9). Furthermore, differentiation of CD4+ T cells into Fox3+ regulatory T cells is also promoted (10). As a result, these coinhibitory pathways have been proven to be the key mechanism for tumor immune evasion as high and sustained PD-1 & PD-L1/L2 expression is often reported in cancer and chronic infections (11, 12). Blocking this pathway has shown improvements in T cells functionality and cancer patients’ survival (13–15). As such, PD-1/PD-L1 interaction has become the corner stone for understanding the physiology of these inhibitory co-receptors in immune system also termed as inhibitory immune checkpoints.

PD-1 and PD-L1 broad range of expression and complex regulation is indicative of its intricate functional domain (12, 16). Recently, its detection in plasma of cancer patients has open a new paradigm for investigations (17–19). Even though, these soluble forms are mainly evaluated for their predictive and prognostic significance, they still retain their inhibitory functionality (18–20). In this review we will elucidate the anti-cancer properties of soluble PD-1, its predictive, prognostic as well as therapeutic value. In order to fully comprehend the role of soluble PD-1, a short review of membrane-bound PD-1/PD-L1 structure, expression, regulation, and mechanism of interaction between PD-1 and PD-L1 is outlined.

## Structure of PD-1

**Figure 1:**
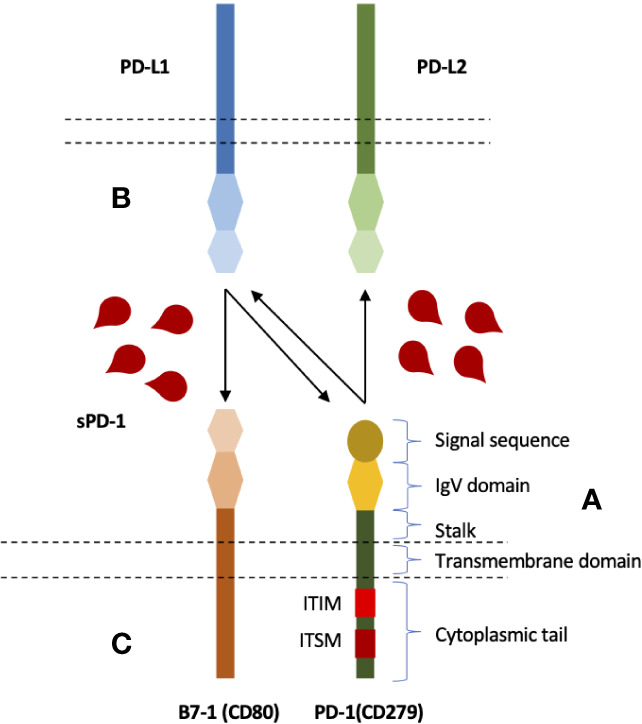
Structure and interactions of PD-1 immune checkpoint molecule, and blocking potential of soluble PD-1. **(A)** PD-1 is composed of an extracellular IgV like domain carrying signal sequence, a transmembrane domain, an intracellular domain that contains two signaling motifs, ITIM and ITSM. **(B)** PD-1 expressed on the surface of T cells (and several other cells) can bind to its ligands; PD-L1 and PD-L2. **(C)** PD-L1, in addition to PD-1, has also been shown to interact with B7-1 expressed on T cells. B7-1 and PD-L1 interactions can also inhibit T cells. Soluble PD-1, therefore, can potentially inhibit all three interactions.

Membrane-bound PD-1 is a type I transmembrane glycoprotein belonging to CD28 family of receptors, a subgroup of immunoglobulin superfamily (21, 22). PD-1 gene was first discovered in T cell hybridoma and hematopoietic progenitor cell line as an upregulated gene (23). PD-1, a 55 kDa glycoprotein of 288 amino acids, is composed of N-terminal IgV like domain that also contain the signal sequence, a 20 amino-acid stalk, a transmembrane domain, and a 95 amino-acid intracytoplasmic domain that contains two tyrosine-based signaling motifs (24) (**Figure 1**). PD-1 IgV-like domain shares about 21% to 33% sequence resemblance with other CD28 family members such as CD28, ICOS, and CTLA-4. PD-1 lacks intracellular SH2 (Src Homology 2) or SH3 binding motifs unlike CD28 and CTLA-4 (25). Its Intracellular domain contains one N-terminal sequence (VDYGEL) constituting an immunoreceptor tyrosine based inhibitory motif (ITIM) and one C terminal sequence (TEYATI) forming an immunoreceptor tyrosine-based switch motif (ITSM) (26). SHP2 recruitment by ITSM phosphorylation results in the inhibitory function associated with PD-1. PD-1 is produced as monomer as it lacks the cysteine residue that is required for homodimerization (21).

## PD-1/PD-L1 Expression and Up-Regulation

PD-1 is up-regulated on T cells after its activation, and is also expressed on other immune cells such as B cells, NK cells, and NKT cells (11, 16, 22, 27). A small portion of myeloid cells, APCs, and innate lymphoid cells (IL2) have also shown PD-1 expression (11, 16, 22, 27, 28). In mouse T lymphocytes, a stronger feedback for T cell mediated immunity was generated with synergistic actions of TCR signals and IFN-α in regulating the expression of PD-1 (29). IFN-α had also exhibited its regulation of PD-1 in macrophages through JAK/STAT signaling pathway (30). IL-6 and IL-12 had also augmented TCR-induced PD-1 expression by activating STAT3/STAT4 (31). In addition, PD-1 expression on T cells can also be induced by cytokines such as IL-2, IL-7, IL-15, and IL-21 (32). Its ligands, PD-L1 and PD-L2, are mainly upregulated on APCs and various cancer cells. PD-L1 expression is diverse and several non-hematopoietic cells have also demonstrated PD-L1 expression such as vascular endothelial cells, non-parenchymal cells of the liver, pancreatic islets, placental syncytiotrophoblasts, keratinocytes and the cornea (11, 16, 22, 27, 28). Regulation of PD-L1 is extensively elaborated particularly in cancer cells as it is the main tactic for immune evasion. PD-L1 regulation in cancer cells involve several extrinsic and intrinsic factors. PD-L1 expression was increased with 9q24.1gene amplification in nodular sclerosing Hodgkin lymphoma and primary mediastinal large B-cell lymphoma (33). Elevated levels of transcriptional factors STAT3 and HIF-1α were also associated with PD-L1 expression in various cancers (34, 35). Oncogenic aberrant signaling pathways such as EGFR, MAPK, PI3k-AKT were shown to upregulate PD-L1 in lung cancers (36–38), various cytokines have shown to mediate PD-L1 expression including IFN-γ, IFN-α, IL-2, IL-6, IL-10, IL-12, IL-15, IL-17, IL-25, IL-27 (32, 36, 39–44). Viral infections such as EBV have also shown to drive PD-L1 expression in gastric and nasopharyngeal cancers (45, 46). Long non-coding RNAs and microRNAs are associated with negative regulation of PD-L1 expression. Long non-coding RNA (lncRNA), NKX2–1, limits lung carcinoma cell migration through negatively regulating PD-L1 expression (47). Similarly, several microRNAs including miR-34a, miR-200, miR-152, miR-155, miR-513, and miR-570 negatively regulate PD-L1 expression in cancers (48–54). PD-L2 on the other hand is mainly expressed by dendritic cells, macrophages, in some mast cells. In addition, it has also been reported to express on some B cells, Th2 cells and few types of cancers (11, 16, 22, 27, 28). Such diverse expression of this checkpoint suggests a broad functional domain (16). Inhibition of T cells primarily through PD-1 interaction with PD-L1 mainly involves two counterparts; antigen presenting cells, and cancer cells. PD-1/PD-L1 interaction leads to a chain of intracellular events in T cells, which ultimately culminate in T cell inhibition and tumor immune tolerance.

## Mechanism of T Cell Inhibition

**Figure 2:**
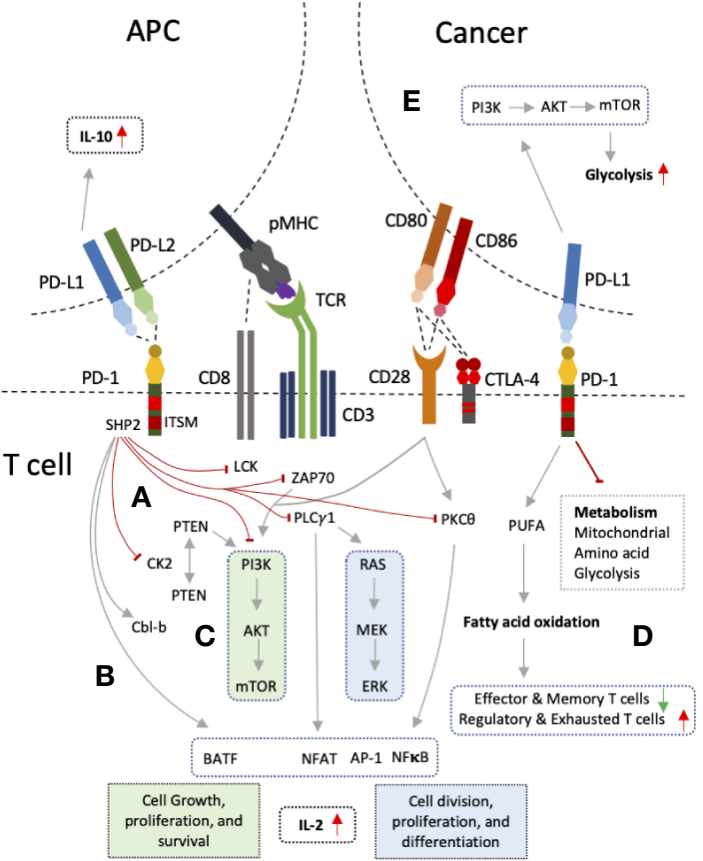
PD-1 and PD-L1 interactions and mechanism of T cell inhibition. **(A)** PD-1 ligation to PD-L1 leads to recruitment of SHP2 by its intracellular signaling motif, ITSM. SHP2 exerts its inhibitory action on proximal signaling molecules of T cell receptor (TCR) such as LCK, ZAP70 resulting PI3K pathway inhibition. PI3K pathway is also inhibited through PTEN by inhibiting CK2. PLC-γ1 and PKCθ are also inhibited results in RAS pathway inhibition, decrease production of IL-2, and reduce expression of several transcription factors such NFAT, NFκB, and AP-1. **(B)** PD-1 also up-regulate Cbl-b and transcription factor BATF. ** (C)** The outcome is inhibition of two main signaling pathways PI3K-AKT-mTOR and RAS-MEK-ERK which results in decreased cell division, growth, proliferation, differentiation, and survival. **(D)** Inhibition of these pathways also lead in altered T cell metabolism with increased inhibition of glycolysis, mitochondrial and amino acid metabolism, and increased fatty acid oxidation. **(E)** Reverse signaling through PD-L1 can also occur in tumor cells and antigen presenting cells (APCs) resulting in PI3K pathway up-regulation and enhanced glycolysis in cancers, and increased IL-10 production in DCs.

Triggering of PD-1 mediated intracellular signaling upon PD-L1 ligation mainly results in the suppression of two main pathways; PI3K-Atk-mTOR, and RAS-MEK-ERK (55, 56) (**Figure 2**). Suppression of these pathways restricts cell growth, differentiation, cell cycle progression, and cell division, proliferation and cell survival. Intracytoplasmic tail of PD-1 contains ITSM motif which recruits SHP-1/2 upon phosphorylation (57). ITSM-recruited SHP-2 results in impaired phosphorylation of ZAP70 and LCK, proximal signaling molecules of TCR, with subsequent inhibition of PI3K-AKT-mTOR pathway (55). In addition, PD-1 was also shown to inhibit downstream signaling to PKC θ (55). Another mechanism by which PI3K-AKT-mTOR pathway is inhibited by PD-1 involves the phosphorylation and phosphatase activity of the PTEN (58–60). PTEN inhibits PI3K pathway while its phosphorylation by CK2 during T cell activation ensues PTEN stability and reducing PTEN lipid phosphatase activity (58, 59). PD-1, on the other hand, disrupts the PTEN stability and increase the phosphatase activity by inhibiting the CK2 mediated phosphorylation (60). PD-1 mediated inhibition of the PI3K pathway suppresses oxygen consumption; affecting T cell glycolytic and glutaminolytic activity, which are up-regulated upon T cell activation (61–64). It inhibits the mitochondrial and amino acid metabolism while beta-oxidation of fatty acids is promoted (64). PD-1 ligation is also associated with polyunsaturated fatty acids accumulation which can suppress T cell immunity (64, 65).

The second major pathway, RAS-MEK-ERK pathway, is disrupted by PD-1 through inhibition of PLC-γ1 (56). Ca^2+^ and DAG (diacylglycerol), downstream of PLC-γ1, activate the RasGRP1 resulting in RAS-MEK-ERK pathway activation (66). Calcium influx also results in dephosphorylation of NFAT and its translocation to nucleus. Cooperation of NFAT with AP-1, one of the three transcription factors (NFAT, NFκB, AP-1) activated after CD28 interaction with its ligands (CD80/86), is required for IL-2 gene transcription and mRNA stability (67). PD-1 ligation has also been reported to up-regulate Cbl-b, which is associated with PI3K inhibition either directly or through suppression of PTEN inactivation (68–70). Besides, Cbl-b also reduces PLC-γ1 activation in anergic T cells (71, 72). On the other hand, PD-1 increases expression of BATF transcription factor, and p38 MAP kinase pathway is left unaffected affecting cell differentiation, apoptosis and autophagy (56, 73). PD-L1 has no such signaling motifs in its cytoplasmic; hence, incapable of carrying out any signaling (74, 75). However, it was shown to function as inhibitory receptor preventing apoptotic death of cancer cells which necessitated the intracellular domain of PD-L1 (74). Furthermore, association of PD-L1 expression on cancer cells with PI3K-AKT-mTOR signaling pathway and enhancement of glycolytic metabolism indicates PD-L1 may also mediate reverse signaling (75). Reverse signaling through PD-L1 expression has also been shown (76). Blocking PD-L1 on DCs with sPD-1 was able to inhibit DCs and increase in IL-10 production (76). Hence, PD-1 pathway essentially down-modulate TCR and CD28 signaling, decreased induction of downstream transcription factors, and decrease cytokine production resulting in decreased T cell proliferation and survival.

## Soluble Production

**Figure 3:**
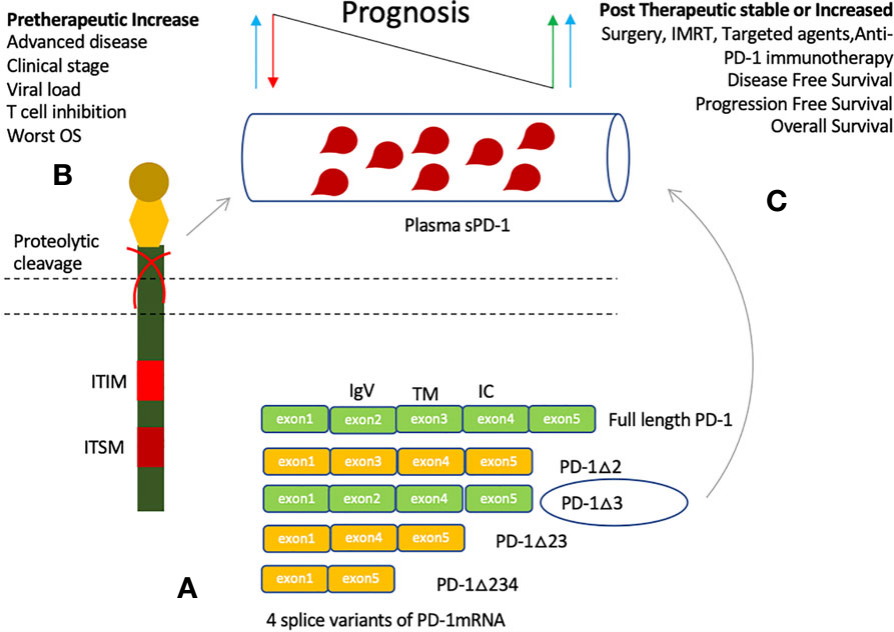
Production of soluble PD-1 and clinical significance of its elevated plasma levels. **(A)** Soluble PD-1 may be produced by proteolytic cleavage or through alternative splicing. Four splice variants are identified, only PD-1△3 may represent the soluble PD-1 as it can bind its ligands. Other splice variants lack exon 2 and hence no extracellular IgV like domain. **(B)** Elevated plasma levels are detected in several cancers, and associated with viral load, cancer risk, clinical stages of cancer, and worst prognosis. **(C)** An increase in sPD-1 levels after induction of therapy have been associated with improved Disease-free survival (DFS), Progression-free survival (PFS), and Overall survival (OS).

Soluble forms of many immune regulatory molecules, both co-stimulatory and co-inhibitory molecules, are detected in plasma of cancer patients including sCTLA-4, sHLA-G, sCD80, sB7-H3, sCD86, sBTLA, sLAG-3, sCD27, s4-1BB, and sCD40 (19, 77, 78). These soluble forms are either produced by shedding off the membrane form or through alternate splice variants (79). Four splice variants of PD-1 have been discovered while sPD-L1 is mainly thought to be produced by proteolytic cleavage of membrane-bound PD-L1 (80). Full length PD-1 (flPD-1) is composed of five exons (exons 1-2), while its fours splice variants arises with the deletion of middle exons 2, 3, and 4 (**Figure 3**). Splice variant PD-1△x2 is generated with deletion of exon 2 corresponding to the extracellular IgV domain; hence, it is unable to bind its ligands. Similarly, PD-1△x2/3 and PD-1△x2/3/4, are also unable to bind its ligands as they have no exon 2. PD-1△ex2,3,4, in addition to exon 2 and 3, also lacks exon 4 that encodes the intracellular domain, and contains a premature stop codon in exon 5. Only, PD-1 △x3 can encode soluble form of PD-1 as it retains the extracellular domain, and only lacks exon 3 corresponding to the transmembrane region of PD-1. Increased level of each PD-1 transcript was seen upon activation of human PBMCs with anti-CD3 plus anti-CD28 monoclonal antibodies. Upon activation, a parallel increase in expression of flPD-1 and PD-1△x3 was observed indicating an important interplay between the soluble and membrane form of PD-1 in maintenance of peripheral self-tolerance and prevention of autoimmunity (80). Reduction of HCC decreased the sPD-1 levels suggesting primary tumor site and tumor-specific T cells as the prime source of circulating PD-1 (81). Low sPD-1 plasma levels were associated with brisk tumor-infiltrating lymphocytes (TILs), moderate levels with non-brisk TILs, and high levels with absent TILs in primary melanoma patients indicating engaged T cells may produce more mPD-1 but low soluble forms and peripheral tumor specific T cells may constitute the main source of elevated PD-1 levels (82). Furthermore, a positive correlation was observed between the levels of sPD-1 and sPD-L1 in advanced pancreatic cancer signifying a common provenience and simultaneous release (83, 84). In cancer patients, studies have reported parallel elevation of both soluble molecules, PD-1 and PD-L1, in plasma suggesting they may have regulatory properties in order to counter the effect of each other as in the case of membrane-bound up-regulation (83–88). Patients with HCC had also observed a positive correlation in the fold-changes of sPD-1 and sPD-L1 at 2 weeks of sorafenib treatment (77). It indicates that a balance between the two molecules may exist locally or in the peripheral circulation as inhibitory signals are mainly required for maintaining balance in the immune system: a state of immune homeostasis (16). Hence, increasing either may impede the function of the other. An increase in sPD-1 may suggest further inhibition of sPD-L1/mPD-L1 thereby avoiding the T cell from inhibition. Similarly, an increase in sPD-L1 may further strengthen T cell inhibition, strengthening cancer immune evasion, and resulting in poor outcome.

## Predictive and Prognostic Significance

Higher sPD-1 levels are predictive of active and advanced disease as well as worst prognosis (81–92). Soluble PD-1 levels association with systemic inflammation markers (CRP), active disease, hepatitis B viral load, HCC (Hepatocellular carcinoma) risk, and worst prognostic factors in DLBCL (Diffuse large B-cell lymphoma) is indicative of its predictive value (83, 84, 89, 90). However, its association with poor prognosis is not fully established. Pretherapeutic increase has been related to worst prognosis in various cancers (81, 84). On the other hand, studies have shown stable or increase in sPD-1 level after induction of cancer therapy was associated with improved outcome such as surgery, IMRT (Intensity-modulated radiotherapy), and EGFR TKIs (Epidermal growth factor receptor tyrosine kinase inhibitors) (86, 88, 91).

### Pretherapeutic Increase

Chronic inflammation, autoimmune disease and cancers are reported to have higher tissue expression and plasma levels of coinhibitory molecules, PD-1 and PD-L1 (81–97). As such, elevated levels of sPD-1 have been detected in several cancers such as NSCLC (Non-small cell lung carcinoma), DLBCL, CLL (Chronic lymphocytic leukemia), NPC (Nasopharyngeal carcinoma), HCC, pancreatic adenocarcinoma, advanced rectal cancer, and metastatic melanoma (81–92) (**Figure 3**). Pretherapeutic higher plasma levels have been associated with disease severity, clinicopathological features, and viral load as well as prognosis (81, 83, 84, 89–91). sPD-1 was detected in 21% of NSCLC patients prior to erlotinib treatment, and in 37% at disease progression (91). Advanced pancreatic cancer patients with elevated levels of systemic inflammation marker, CRP, had a trend towards higher mean sPD-1 levels; yet, no association for higher sPD-1 levels with worst survival was observed (83). However, in a separate study of 32 pancreatic adenocarcinoma patients, high sPD-1 levels were associated with worst survival (84). HBV (Hepatitis B virus) patients had higher sPD-1 plasma levels in comparison to HBV resolvers and healthy controls (81). Higher sPD-1 levels were indicative of HBV viral activity and HCC risk (89). HBV-related HCC had higher sPD-1 levels as compared to other HBV clinical diseases, and was associated with poor survival (81). In retrospectively analyzed 120 HCC patients treated with radical resection, higher sPD-1 level was associated with improved disease-free survival (DFS) and overall survival (86). In patients with DLBCL, elevated pretherapeutic sPD-1 levels were associated with worst prognostic indicators such as age (≥60), disseminated clinical stage, performance status (ECOG ≥2), elevated levels of plasma LDH, and presence of ≥1 extranodal site (90). sPD-1 levels have also been revealed to be higher in certain genotype or phenotype of disease. HBV genotype C, and non-GCB phenotype of DLBCL cancer patients had higher sPD-1 levels (89, 90). These outcomes suggest that sPD-1 levels are detected in patients with chronic infections and cancer, and may increase at disease progression. However, depending on the treatment undertaken, it may or may predict survival outcome.

### Post-Therapeutic Increase

On the other hand, post therapy sPD-1 levels’ stability or increase have demonstrated a distinct behavior. No significant increase in sPD-1 levels were observed after induction of neoadjuvant chemoradiotherapy in advanced rectal cancer patients, and no association with survival was established (92). NSCLC patients treated with EGFR TKIs, however, were revealed to have increased the sPD-1 levels which was associated with improved progression free survival and overall survival (91). Recently, studies have reported enhancement of immunogenicity and clinical response after anti-EGFR mAb cetuximab or nimotuzumab through STAT1-induced HLA class I upregulation (98, 99). As HLA-I upregulation is associated with tumor-specific CD8+ mediated immune response, these cells may also lead into sPD-1 secretion after anti-EGFR treatment. Increase in sPD-1 was associated with a decrease plasma EBV-DNA level and improved survival in patients with nasopharyngeal carcinoma patients after Definitive Intensity-Modulated Radiotherapy (IMRT) (88) (**Figure 3**). One may speculate that the increase in sPD-1 levels may indicate radiotherapy induced immunity as radiotherapy is associated with increase in antigen presentation resulting in increased amount of tumor-specific T lymphocytes leading to an increase in soluble PD-1 production (100, 101). PFS and OS was also improved for NSCLC patients with a stable or increase in sPD-1 after undertaking two cycles anti-PD-1 mAb therapy (nivolumab) (87). The underlying mechanism for sPD-1 increase after certain treatments (radiotherapy, anti-EGFR therapy, and anti-PD-1 immunotherapy) has not been investigated (87, 88, 91). It may indicate re-activation of tumor-specific cytotoxic T lymphocytes as these cells make up the primary source of sPD-1 in circulation (81, 83, 84). Re-activation can result partly from improving antigen presentation by APCs, upregulation of HLA-I on tumor cells, and alleviating T cells’ inhibition after treatments such as radiotherapy, anti-EGFR mAbs/molecular agents, and anti-PD-1 immunotherapy (87, 88, 91, 98, 99). Furthermore, increase in sPD-1 levels after treatment was associated with improved outcomes in terms of decreasing plasma EBV-DNA level, and improving PFS and OS (87, 88, 91). It indicates increased sPD-1 level represents immunogenicity and that sPD-1 is biologically active. As our understanding broadens about the underlying mechanism of efficacy of such treatments, we may better analyze the mechanism of sPD-1 increase and its role in improving the outcome of cancer patients.

## Anti-Cancer Activity

**Figure 4:**
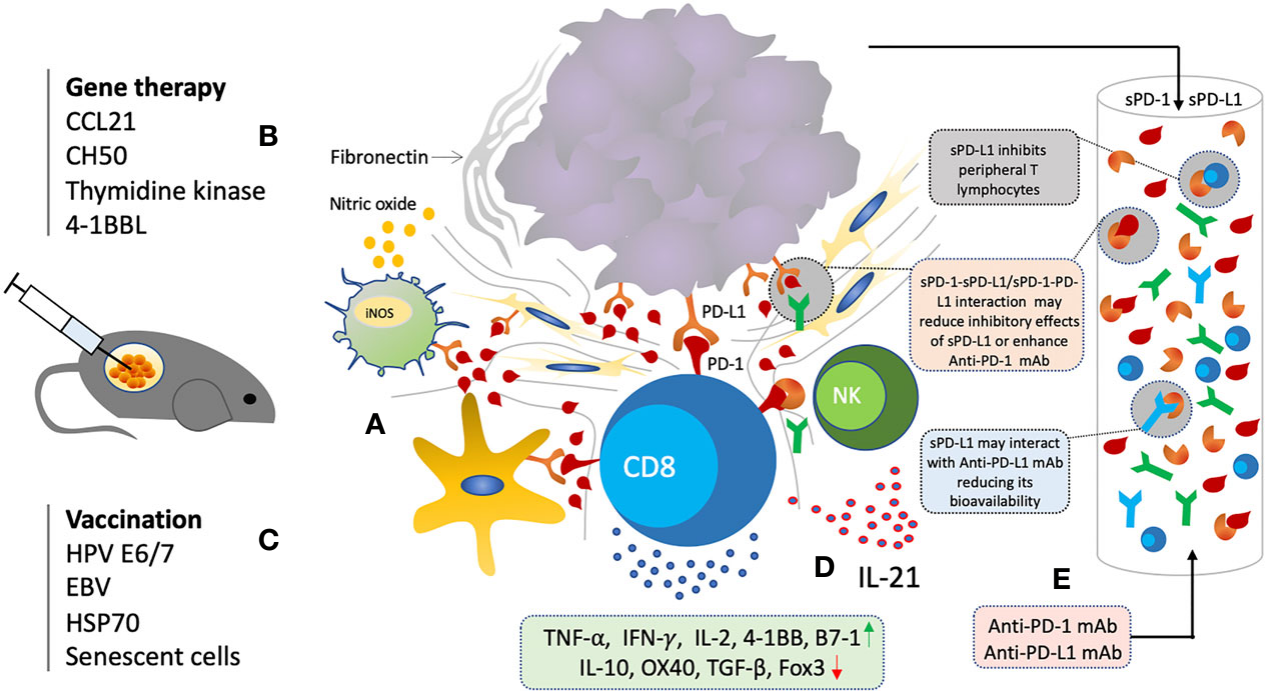
Soluble PD-1 based therapeutic strategy. **(A)** Local gene delivery of sPD-1 induce anti-cancer immunity and reduction in tumor growth through interruption of PD-1/PD-L1 interactions in TME. **(B)** Combination of sPD-1 with other gene therapeutic agents targeting cancer cell survival, chemotaxis, and costimulatory molecules have shown synergistic activity **(C)** Combination of sPD-1 also enhance vaccine induced immunity and overcome vaccine resistance. **(D)** Combination of sPD-1 also enhance IL-21 induced immunity through increase infiltration and cytotoxicity of CTLs and NK cells **(E)** Elevated levels of sPD-1 and sPD-L1 may have regulatory functions and interfere with anti-PD-1/PD-L1 mAb based immunotherapy.

Soluble PD-1, just like membrane-bound PD-1, is biologically active and can inhibit mPD-1/PD-L1 and mPD-1/PD-L2 interactions (102–105). sPD-1 blockade has shown to regulate T cell functions through PD-L1/2 blockade in various autoimmune diseases, chronic infections, and antitumor immunity (102–107). Several animal studies have evaluated the anti-cancer effects of sPD-1 though local gene transfer using eukaryotic expression plasmid and other viral vectors (102–105, 107) (**Figure 4**). Increase in activation and cytotoxicity of T cells, and reduction in tumor growth was observed through *in vitro* and *in vivo* blockade of PD-L1 by sPD-1 transferred *via* eukaryotic expression plasmid (102–105). *In vitro* blockade of PD-Ls expressed on H22 cells showed enhanced tumor cell lysis by HSP70-peptide complex-stimulated spleen cells (103). H22 cells and spleen cells expressing both PD-L1 and PD-L2 were also shown to be inhibited by secreting sPD-1 plasmid transfected cells (103). In animal model of BALB/c mice inoculated with H22 hepatoma, not only rate of tumorigenesis was slowed but also lower number of mice had displayed tumorigenesis injected with pPD-1A (103–105). Moreover, inhibitory effect of sPD-1 on tumor was similar to that of mice injected with anti-PD-L1 mAb (103). Similarly, reconstructed adeno-associated virus plasmid encoding sPD-1 was also shown to induce anti-tumor immunity (107). sPD-1 was able to regress tumor and prolong survival of tumor bearing mice (107). sPD-1 treated mice exhibited tumor-specific cytotoxic T cells infiltration (107). IL-10 pretreated DCs with up-regulated PD-L1 and decreased co-stimulatory ability for lymphocytes activation showed enhanced lymphocytic activation after co-culturing with pPD-1A-transfected BHK cells (103). Enhanced cytotoxicity was observed even when the H22 cells were pretreated with sPD-1 indicating that sPD-1 inhibiting the PD-Ls present on DCs resulting in T cell activation (103–105). Splenocytes were revealed to have significantly increased mRNA expression of IFN-γ with moderate increase in TNF-α, 4-1BB, and B7-1, while that of OX40 and IL-10 were downregulated (103). Such panel is indicative of CD8+ T cells activation probably through 4-1BB/4-1BBL and B7-1. Down-regulation of OX40 and IL-10 suggests that the CD4+ T cells may not play any critical role in sPD-1 dependent antitumor immunity improvement. Membrane-bound PD-1 blockade has also shown a similar augmentation of Th1/Th17 response with enhanced production of IFN-γ, IL-2, TNF-α, IL-6, and IL-17A, and reduction of Th2 cytokines IL-15 and IL-13 (108). Likewise, soluble PD-1 was also shown to aggravate the progression of collagen-induced arthritis through Th1 and Th17 pathways (109). Overall, sPD-1 is shown to increase anti-tumor immunity through *in vitro* and *in vivo* blockade of PD-L1 and PD-L2 present on DCs and cancer cells with an increase in activation, cytotoxicity, cytokine production, and infiltration of CD8+ T cells.

Reverse signaling through sPD-1 interactions with PD-L1 and PD-L2 expressed on DCs has also been demonstrated resulting in decreased OVA-specific CD4+ T cells and inefficient DC maturation at low doses of antigen (76). However, increased T cell proliferation was shown at higher antigen concentration indicating sPD-1 effects may differ depending on the strength of TCR signaling and degree of DC maturation. Such reduced DC maturation and increased apoptosis was also revealed in HIV infected individuals with an *in vitro* PD-L1 engagement of sPD-1 protein on myeloid DCs (110). On the other hand, *in vivo* codelivery of sPD-1 DNA with DNA vaccine demonstrated enhanced DC maturation probably indirectly through blockade of signals delivered by PD-1 on T cells (111). Several other reports have also shown such T cell activation mediated DC maturation (112, 113). In addition, PD-L1 has also been reported to interact with B7-1 and inhibit T cell activation and cytokine production (114). Hence, sPD-1 can also inhibit PD-L1:B7-1 interactions in addition to its ligands, PD-L1 and PD-L2.

Soluble PD-1 had also shown enhancement of the anti-tumor effects induced by other gene-therapeutic agents such as secondary lymphoid tissue chemokine (SLC, CCL21), Herpes Simplex Type 1 Thymidine Kinase (TK) (HSVtk), a recombinant fibronectin (CH50), and 4-1BB ligand gene, mainly through strengthening CD8+ T cell responses resulting in tumor growth reduction (104, 115–118) (**Figure 4**). Anti-tumor immunity induced by several vaccine agents were also improved with sPD-1 delivery such as HSP70 vaccine, and human papilloma virus-16 E7 DNA vaccines (111, 119, 120). Furthermore, it had also been able to enhance the T cell and NK cell immunity induced by IL-21 through blocking the PD-1/PD-L1 interaction pathway (121). Such broad scope potential of sPD-1 local gene delivery indicates its great potential for anti-cancer therapy.

## Implications for Anti-Cancer Therapy

*In vitro* blockade of PD-L1 and PD-L2 has been demonstrated with sPD-1 through local gene transfer (102–105). sPD-1 through binding to mPD-L1/L2, its interaction with these ligands could prevent the mPD-1 on T cells from binding thereby avoiding T cell inhibition. Recently, PD-L1 has also been reported to bind to B7-1 (CD80) resulting in T cell inhibition in an indirect manner by preventing CD80:CD28 interaction (114). Hence, in addition to blocking PD-1/PD-L1 and PD-1/PD-L2 interactions, sPD-1 may also interrupt PD-L1/B7-1 interaction by binding to PD-L1 (**Figure 1**). Furthermore, it may also block the inhibitory action of circulating sPD-L1 as it has been associated with worst survival and impediment to ICB (immune checkpoint blockade) (17, 122). Therefore, increasing the sPD-1 level locally or systematically may have several implications for cancer immunotherapy.

### Reducing Inhibitory Effects of Circulating Soluble PD-L1

An enormous amount of clinical research has been going on in the last few years about the predictive, prognostic, and regulatory functions of circulating PD-L1 levels. Circulating PD-L1 is biologically active and has been shown to inhibit T cell functions in various cancers (20, 123, 124). Higher plasma levels have been well correlated with disease status, severity and prognosis (83–88, 92, 125, 126). Elevated levels of circulating PD-L1 have also been shown to predict various treatments’ efficacy such chemotherapy, radiotherapy, and immunotherapy (17, 122, 127–134). As described earlier, plasma levels of sPD-1 produced by tumor-specific T lymphocytes and sPD-L1 secreted by cancer cells have shown proportionality, suggesting a state of immune homeostasis that is favoring tumor cells (83–88). Hence, hypothetically, increasing the sPD-1 levels which can bind to these circulating PD-L1 as well as mPD-L1 may essentially lessen T cell inhibition (**Figure 4**). As such, sPD-1 delivery holds great potential as an anti-cancer strategy to alleviate T cell inhibition. Furthermore, sPD-1 delivery in combination with these treatments may also enhance their anti-cancer efficacy.

### Soluble PD-1 as Alternative to mAb-Based Immunotherapy

*In vitro* and *in vivo* inhibitory effects of sPD-1 shows it can serve a similar function to anti-PD-1 or anti-PD-L1 monoclonal antibodies thereby preventing PD-L1 or PD-L2 from binding to mPD-1 present on T cells. As such, *in vivo* inhibitory effects on tumor growth was revealed to be similar to that of anti-PD-L1 mAb injected mice (103–105). Besides, it can block all three interaction as compared to dual targeting anti-PD-1 and anti-PD-L1 mAb thereby enhancing the T cell immunity in direct and indirect manner through blocking B7-1/PD-L1 interaction (114). Additionally, local gene therapy, in general, would have some advantages over antibody-based therapy (135–137). For example, sustained expression of genes would make it cost-effective as compared to frequently required high dose injections of mAb (135, 136). Similarly, it was found that dosage would not raise concerns about toxic side effects associated with systemic administration of Abs (137). Altogether, advantages of gene-based therapy combined with anti-cancer potential of sPD-1 make this anti-cancer therapeutic strategy more desirable.

### Soluble PD-1 in Combination With Other Therapies

Cancer immune evasion involves development of an immune suppressive tumor microenvironment, reduced immune recognition, increased resistance and survival (138). Hence, therapies could be combined aiming at different aspects of cancer immune evasion. Immune checkpoint blockade has shown limited success due to reinvigoration of existed T cells. Therapies aimed at increasing antigen production, release and presentation would increase recognition such as RT (139). In addition, vaccination aimed at existed tumor mutations or cytokine therapy are often faced with resistance through up-regulation of immune inhibitory checkpoint molecules including PD-1/PD-L1 pathway (111, 119). Several animal studies have demonstrated sPD-1 local gene therapy delivery further improving anti-cancer immunity induced by other anti-cancer therapeutics when given in combination or to overcome therapy/vaccine-induced immune resistance (104, 115–118). We will review all various types of combination strategies that could potentially increased sPD-1 anti-cancer effects.

#### Gene Therapy

Several factors that can disrupt the cancer immunosuppressive microenvironment may hold potential for anti-cancer gene therapy. Chemokines makes up an important entity of whole immune process particularly in recruiting the immune cells (140). A secondary lymphoid chemokine, CCL21, was evaluated using eukaryotic expression plasmid for local gene therapy (104). *In vitro* chemokine (pSLC) delivery had induced spleen T cell migration and *in vivo* tumor growth inhibition. However, PD-L1, PD-L2 gene expression in tumor cells was observed, and expression was increased with increasing dose of pSLC. Administration of sPD-1 expressing eukaryotic expression plasmid improved the cytotoxicity of tumor-specific CTLs induced by pSLC. Combinatorial local gene transfer was associated with enhanced inhibition of tumor growth with no evidence of autoimmunity.

Similarly, Fibronectin (matrix glycoprotein), an essential component of extracellular matrix is aberrantly expressed in several cancers facilitating tumor growth, invasiveness and metastasis, and resistance to therapy (141). CH50, a recombinant polypeptide with bifunctional domains (CellI and HeparinII), was shown to inhibit tumor growth, metastasis, and regulate macrophages by down-regulation of CDC2, αvβ3 integrin, MMP-2/9 in the tumor microenvironment (142, 143). Qiu et al. had constructed a sPD-1-CH50 (a recombinant polypeptide with 3 functional) for anti-cancer immunity evaluation in PD-L1 expressing tumor cells (116, 117). sPD-1-CH50 had successfully intensified the cytotoxicity of macrophages and CTLs through iNOS, TNF-a, and IFN-γ. *In vivo* restriction of hepatoma growth and invasiveness was also demonstrated (116). Inducing thymidine kinase expression in cancer cells through adenoviruses harboring herpes simplex virus thymidine kinase gene (HSVtk), and exposing these expressing cells to ganciclovir (GCV) results in a series of phosphorylation that ultimately leads in cancer cell apoptosis (144–147). *In vivo* and *in vitro*, it has shown ant-tumor activity probably through improved antigen presentation, which was further enhanced by sPD-1 *via* enhancing the CD8+ T cells responses (115). Systemic effects of this localized gene delivery were also observed in tumor bearing mice. HSVtk has shown its potential for gene therapy in brain cancer in clinical studies (148). Though, single HSVtk gene therapy has failed to show effectiveness in larger Phase III trials in GBM patients, its potential for combinatorial gene therapy still needs to be evaluated (149, 150). HSVtk and FMS-like tyrosine kinase 3 (FMStk-3, a DC growth factor), in combination had demonstrated anti-tumor adaptive immune responses and cancer cell death in GBM (151). *In vivo* somatic GM-CSF, IL-2, and HSVtk combination gene therapy had also developed anti-tumor immunity against non-immunogenic mammary carcinoma (152). Therefore, adding sPD-1 can further enhance the therapeutic effects of these agents in brain cancers as well as other cancers.

#### Cancer Vaccination

Several vaccines strategies have been employed against cancer antigens using whole tumor lysates, synthesized peptides, viral vectors with tumor antigens expression, dendritic cells-based vaccination (153). Virus-related cancers such as Human papilloma virus and Epstein-Barr virus have been considered ideal for immunotherapy as foreign antigens are consistently present (154). HPV proteins, E6 and E7, have been used for anti-therapeutic vaccines due to their carcinogenicity and foreignness (155, 156). HPV therapeutic vaccines are in clinical development for HPV-related precancers cervical intraepithelial neoplasia (CIN) and vulvar intraepithelial neoplasia (VIN) (157). In addition, HPV also have causative association with penile, anal, vulvar, vaginal, oropharyngeal, and laryngeal precancers or cancers (158). Their efficacy has been demonstrated in HPV-related precancers in comparison to cancers (159). It could be due to several operative immunosuppressive forces including PD-1 and PD-L1 interactions as HPV was itself related to PD-1 and PD-L1/L2 upregulation in HR-HPV+ CIN 1 and CIN 2/3 and metastatic cervical cancer (160). Hence, several animal studies have evaluated the combination of ICBs and E6/E7 vaccine combination resulting in increased CD8+ TILs and suppression of tumor growth (161–165). Soluble PD-1 delivery had also shown enhancement of primary and memory CD8+ T cells mediated anti-tumor immunity induced by human papilloma virus-16 E7 DNA vaccine (111). Furthermore, *in vivo* maturation of OVA-pulsed BM-DCs cocultured with activated T cells was accompanied by up-regulation of DC maturation markers (CD86, CD40, and MHC-II) after being treated with sPD-1 (111). It indicates immunization strategy to increase antigen-specific T cell immunity induced by vaccination with sPD-1 as an adjuvant.

Therapeutic EBV vaccines have been designed and tested in nasopharyngeal carcinoma (NPC) mainly aimed at EBNA1, LMP2, and have shown immunogenicity by inducing activated CD4+ and CD8+ T cells responses (166). Though, no study has evaluated the use of such vaccines in combination with sPD-1; an inverse proportional relation was observed between sPD-1 and EBV DNA levels in NPC patients upon IMRT induction (88). After induction of IMRT, survival of patients was prolonged in those who had observed an increase in sPD-1 levels and a decrease in EBV DNA level. The study did not reveal any direct action of sPD-1 on EBV DNA but one may speculate that disrupting the PD-1 pathway may have enhanced the immunity.

Other than viral-related cancers, several other tumor-associated antigens have been evaluated for cancer vaccination such as heat shock proteins (HSP70). HSPs are implicated in cancer growth and resistance to chemotherapy (167, 168). HSP70 and HSP90 vaccines have been evaluated in various trials for its anticancer effectivity in breast cancer, cervical cancer, ovarian, melanoma, renal cell carcinoma, and GBM (169–172). HPS vaccines induced anti-cancer immunity involves the DC maturation, CD8+ and CD4+ T cell-mediated responses, and enhancement of NK cell cytotoxicity (173–180). In an animal study, HSP70 vaccine had induced T cell infiltration, expression of IFN-γ and IL-2, and delaying the pulmonary metastases of melanoma cancer (119). However, an increase in PD-L1 expression was observed in residual tumor cells associated with tumor progression. Addition of sPD-1 successfully overcome the HSP70 vaccine resistance through enhanced expression of IFN-γ and IL-2 genes in tumor-infiltrating lymphocytes and decreased expression of negative regulatory molecules including IL-10, TGF-b, and foxp3. In a similar study, HSP70 vaccine was shown to promote PD-1 and PD-L1 expression in addition to tumor specific cytotoxic T lymphocytes. The combination of sPD-1 and HSP70 further potentiated the immune response. The anti-tumor response of the combination was stronger than each agent alone (120). HSP90 proteins are implicated as oncogenic drivers and its inhibition is sought for anti-cancer therapy (181). Combining HSP90 inhibition with immunotherapy, in particular checkpoint blockade, have also been proposed as they have been associated with induction of PD-1 and its ligands expression (35, 182). The combination of a HSP90 inhibitor and an anti-PD-1, PD-L1, and CTLA-4 antibodies have already demonstrated anti-tumor efficacy in preclinical and CRC clinical trials (182, 183). Therefore, sPD-1 delivery along with HSP70/90 vaccines or HSP90 inhibitors holds potential for clinical efficacy.

Recently, senescent cells were used as vaccines as these cells cannot proliferate but still remains metabolically active (184, 185). Accumulation of senescent cells with various SASP (senescence-associated secretory phenotype) could cause proinflammatory microenvironments (185). Nonetheless, the response was restricted by PD-L1 expression. Senescent tumor cell vaccine (STCV) and sPD-1 combination was demonstrated to provide complete protection from 4T1 tumor challenge in mice with pre-injections. Moreover, therapeutic efficacy was also shown by delaying tumorigenesis and suppressing tumor progression at early stages.

#### T Cell Co-Stimulation

Anti-cancer immunity by sPD-1 delivery had shown up-regulation of costimulatory molecules on splenocytes such as 4-1BB, B7-1, B7-2, and CD40 (103, 111). Qiu, et al. injected hepatoma inoculated mice with combinatorial gene for 4-1BB ligand and sPD-1 expression (186). Combined gene expression was associated with enhancement of CD8+ T cells infiltration, greater tumor growth inhibition and improvement in survival of tumor bearing mice (107, 186). The observed effect was superior to each gene-effect alone (107). B7-1 upregulation also indicates it may play a similar role and enhancing the receptor for it could result in synergistic response (103). Soluble PD-1 was able to induce DC maturation and expression of B7-2 and CD40 in the presence of DNA vaccine-induced activated T cells (111). B7-2 is a ligand for costimulatory receptor CD28 on T cells while CD40, a DC surface receptor for ligand (CD40L) is expressed on T cells, is essential for DC-mediated T cell activation. Both of these surface molecules play a critical role in T cell activation. As such, these molecules represent potential targets for gene therapy, much in the same manner as 4-1BB, in combination with sPD-1 delivery. Investigation with a greater panel of immune checkpoint modulators during sPD-1 mediated immunity may reveal further therapeutic options for anti-cancer therapy.

#### Cytokine Therapy

Cancer cytokine therapy has been developing as more and more agents are being investigated for their clinical efficacy as single agent or in combination with vaccines, immune checkpoint inhibitors or agonist antibodies, and adoptive cell therapy (187). These include IFN-α, IFN-γ, IL-2, IL-7, IL-12, IL-15, IL-21, and Granulocyte-macrophage colony-stimulating factor (GM-CSF). They have shown anti-tumor responses in animal models as well as clinical trials by stimulating effector cells including T cells and NK cells. Several of these cytokines were shown to up-regulate PD-1 on T cells such as IL-2, IL-7, IL-15, and IL-21 (32). Cytokines are also associated with up-regulation of PD-L1 with the exception of IL-21. Pan, et al. evaluated the effects of sPD-1 combined with IL-21 therapy in a hepatoma murine model (121). The anti-cancer immunity was enhanced by the combined treatment and significantly inhibited the tumor growth in comparison to IL-21 alone. Enhancement was demonstrated in form of increase in CTL cytotoxicity, numbers of CTLs and NK cells, and upregulation of cytokines such as IFN-γ and IL-2 with downregulation of IL-10.

#### PD-L1 Expression Regulating Molecules

PD-L1 expression is regulated on tumor cells at many levels including genomic, transcriptional, epigenetic, and translational level (188, 189). These regulators include cytokines, intracellular oncoproteins, and microRNAs that regulate PD-L1 regulation positively or negatively in a direct or indirect manner (188, 189). For-example, it has been acknowledged that PD-L1 was upregulated by p53 *via* miR-34 (54). Such a combination of PD-L1 upregulation and miR-34 down-regulation revealed in cervical cancer was exploited by Qin et al. for synergistic anti-cancer responses (190–192). Qin, et al. prepared Cationic lipid microbubbles (CLMBs) loaded with sPD-1 and miR-34a in combination with ultrasound targeted destruction (192). Ultrasound-mediated co-delivery of sPD-1 and miR-34a successfully decreased tumor volume and weight by inducing apoptosis as revealed by downregulation of suppressor gene Bcl-2 and upregulation of proapoptotic gene Bax. Antitumor immunity-related IFN-γ was strongly upregulated and percentage of CTL was also increased. On the other hand, tumor deficient in PD-L1 expression contribute to “target missing” resistance (193). In this sense, positive PD-L1 regulators (epigenetic or some treatments like RT), through upregulation of PD-L1, may be used in combination with ICB or sPD-1 for synergistic anti-tumor effect.

#### Immune Checkpoint Blockade Immunotherapy

Monoclonal antibody-based immune checkpoint blockade has emerged as an attractive, promising anti-cancer strategy aimed at enhancing anti-cancer T lymphocytes (194, 195). The clinical results from the already FDA-approved ICB agents including ipilimumab (anti-CTLA-4 mAb), nivolumab, pembrolizumab (anti-PD-1 mAbs), atezolizumab, avelumab, and durvalumab (anti-PD-L1 mAbs) are unprecedent and encouraging (194, 195). Though, both checkpoint blockades (anti-CTLA-4 and PD-1/PD-L1) rescues anti-tumor T lymphocytes, they are thought to affect different phases of anti-tumor T cell responses (194). Checkpoint blockade of CTLA-4 supports induction phase while PD-1/PD-L1 blockade maintains effector phase of anti-tumor cell responses (194). Therefore, in various cancers, combination of these ICB agents have already been approved (advanced melanoma, renal cell carcinoma) or investigated (NSCLC, recurrent SCLC) for clinical synergy (196–200). Soluble PD-1 gene therapy, in the same manner, may also finds a natural partner in anti-CTLA-4 agents for synergistic immune responses. Delivery of sPD-1 along with anti-PD-1/PD-L1 mAbs may show synergistic effects. As direct local tumor delivery may further enhance anti-tumor response due to elevation of local levels of sPD-1 as PD-1 mediated inhibition strength differentially affects T cell effector functions (45). Simultaneously, higher doses of mAb could be prevented to reach greater efficacy as higher systemic doses are associated with increased side effects. Circulating sPD-L1 plasma level is associated with cancer prognosis, and has been shown to inversely affect the efficacy of anti-PD-1 (nivolumab) mAb in cancer patients (122, 128, 129). Higher sPD-L1 levels were associated with lower nivolumab efficacy and worst prognosis (17, 122). Hence, administration of sPD-1 may interfere with the circulating sPD-L1 thereby reducing its inhibitory action and enhance efficacy of anti-PD-1 mAb. As such, post-therapy increase in sPD-1 level was associated improved survival after two cycles of anti-PD-1 mAb (87). On the other hand, sPD-1 and anti-PD-L1 mAb have the same targets (mPD-L1 and sPD-L1) thereby sPD-1 delivery could increase its efficacy and reduce toxicity associated with mAbs by reducing doses of mAb injections (127). Scope of immune checkpoints is quickly expanding as newer checkpoints such as TIGIT, TIM-3, LAG-3, VISTA, BTLA, B7-H3 etcetera, are being discovered and some of them (TIGIT, TIM-3, and B7-H3) are expressed in combination with PD-1 regulating not only T cells but also NK cells (201–210). These checkpoints also hold potential for combination ICB strategy with sPD-1.

#### Other Conventional Anti-Cancer Therapies

Radiotherapy induces anti-tumor immune response through several mechanisms such as increase in pro-inflammatory cytokines, antigen release from irradiated cells to prime tumor-specific T cell responses, enhancing T cell infiltration into tumors (100, 101, 188, 189). Yet, these anti-tumor immune responses may be blunted by several mechanisms including regulatory T cells and checkpoint molecules such as PD-L1 that promote T cell tolerance and exhaustion. Radiotherapy plus immunotherapy has been revealed in several studies to have synergistic efficacy in treating cancer patients (211, 212). Chemotherapy has also been revealed to reduce regulatory T cell activity, increase ratio of CTL to Tregs, inhibits myeloid-derived suppressor cells, increase antigen presentation, and up-regulate PD-L1 expression (213–218). And so, it has been combined with ICB in lung cancer and revealed to have better efficacy (219). Plasma levels of PD-L1 has also been associated with prognosis in cancer patients receiving radiotherapy or chemotherapy (130–134). Therefore, sPD-1 delivery could have synergistic ant-cancer effects in combination with these treatments.

## Conclusions

Blockade of PD-1/PD-L1 inhibitory immune checkpoint have revolutionized the cancer therapy paradigm; hence, this particular checkpoint pathway has been the subject of intense investigations. Expression, regulation, and interaction of PD-1 and its ligands have enhanced our understanding of how costimulatory and coinhibitory molecules have been exploited by cancer cells for immune evasion. In recent times, a surge in investigations of soluble forms of these molecules detected in the plasma of cancer patients have been observed. These investigations have been mainly focused on the predictive and prognostic values of these molecules. Several studies have revealed that both, sPD-1 and sPD-L1, are elevated in cancer patients and may predict worst prognosis. Production of both molecules is correlated during the course of the disease and may have same provenience. Soluble forms may be produced in order to maintain peripheral self-tolerance and immune evasion. Disrupting this balance by increasing the amount of sPD-1 or sPD-L1 may shift immune balance; enhancing T cell activation through disrupting PD-1/PD-L1 interaction by sPD-1, or T cell inhibition through increased expression of sPD-L1. Such effects are observed in clinical studies. Such as, therapeutic intervention may increase or decrease the level of each molecule, in particular, post therapeutic increase in sPD-1 levels was associated with improved outcome. Also, elevated levels of sPD-L1 are associated with worst outcome and decreased efficacy of anti-PD-1 mAb (17, 122). While increase in sPD-1 was associated with better efficacy of anti-PD-1 mAb (nivolumab) (87). As sPD-1 retain its ability to bind its ligands can, therefore, interrupt PD-1/PD-L1 pathway. As such, sPD-1 may be used as a therapeutic strategy for interrupting PD-L1 interactions in the same manner as monoclonal antibodies. Several *in vitro* and *in vivo* studies have shown that increasing sPD-1 in tumor microenvironment can successfully induce anti-tumor immunity and reduce the cancer growth. Furthermore, it has also shown synergy with several other anti-cancer therapeutic strategies in the same manner as ICB. Also, it can enhance the potential of cancer vaccination and overcome its resistance. It can also be used to overcome cancer therapy resistance as PD-L1 up-regulation is one of the resistance mechanisms implied by cancers. So far, only animal studies have evaluated its candidacy for anti-cancer therapy potential. Given its broad anti-cancer therapeutic potential, it might be the time, the application of sPD-1 as an anti-cancer therapy be evaluated in clinic.

## Author Contributions

All authors have contributed equally. All authors contributed to the article and approved the submitted version.

## Funding

This work was supported by the Natural Science Foundation of Shenzhen (No. JCYJ20170307095828424), Shenzhen Health and Family Planning System Research Project (No. SZBC2017024), and the technical research and cultivation project for the youth of Shenzhen People’s Hospital (No. SYKYPY2019029).

## Conflict of Interest

The authors declare that the research was conducted in the absence of any commercial or financial relationships that could be construed as a potential conflict of interest.
